# A comparative analysis of chiropractic and general practitioner patients in North America: Findings from the joint Canada/United States survey of health, 2002–03

**DOI:** 10.1186/1472-6963-6-49

**Published:** 2006-04-06

**Authors:** Eric L Hurwitz, Lu-May Chiang

**Affiliations:** 1 Department of Public Health Sciences and Epidemiology, John A. Burns School of Medicine, University of Hawaii – Manoa, 1960 East-West Road, Biomed. D-104H, Honolulu, Hawaii 96822, USA; 2 Department of Epidemiology, University of California – Los Angeles (UCLA) School of Public Health, Los Angeles, California, USA

## Abstract

**Background:**

Scientifically rigorous general population-based studies comparing chiropractic with primary-care medical patients within and between countries have not been published. The objective of this study is to compare care seekers of doctors of chiropractic (DCs) and general practitioners (GPs) in the United States and Canada on a comprehensive set of sociodemographic, quality of life, and health-related variables.

**Methods:**

Data are from the Joint Canada/U.S. Survey of Health (JCUSH), 2002–03, a random sample of adults in Canada (N = 3505) and the U.S. (N = 5183). Respondents were categorized according to their pattern of health-care use in the past year. Distributions, percentages, and estimates (adjusted odds ratios) weighted to reflect the complex survey design were produced.

**Results:**

Nearly 80% of respondents sought care from GPs; 12% sought DC care. Compared with GP only patients, DC patients in both countries tend to be under 65 and white, with arthritis and disabling back or neck pain. U.S. DC patients are more likely than GP only patients to be obese and to lack a regular doctor; Canadian DC patients are more likely than GP only patients to be college educated, to have higher incomes, and dissatisfied with MD care. Compared with seekers of both GP and DC care, DC only patients in both countries have fewer chronic conditions, take fewer drugs, and have no regular doctor. U.S. DC only patients are more likely than GP+DC patients to be uninsured and dissatisfied with health care; Canadian DC only patients are more likely than GP+DC patients to be under 45, male, less educated, smokers, and not obese, without disabling back or neck pain, on fewer drugs, and lacking a regular doctor.

**Conclusion:**

Chiropractic and GP patients are dissimilar in both Canada and the U.S., with key differences between countries and between DC patients who do and do not seek care from GPs. Such variation has broad and potentially far-reaching health policy and research implications.

## Background

Chiropractic is the most commonly used unconventional therapy in Canada and the United States, both in the total populations and among primary care and family practice patients [1]. Back pain is one of the leading reasons for seeking general medical care and the top reason for visiting a chiropractor [2,3]. About 20% of the U.S. adult population has ever used chiropractic care, most commonly for low-back or neck pain [3]. In fact, the majority of all health-provider visits for low-back pain are to chiropractors, with patients reporting high levels of satisfaction and helpfulness [4,5]. However, little is known about the similarities and differences of persons who integrate chiropractic care with general medical care vs. those who consult chiropractors alone, either for musculoskeletal or neuromusculoskeletal problems, primary health care, disease prevention, or other reasons.

In recent studies of family practice and primary care patients, 20 to 30% report using some form of complementary or alternative medical (CAM) therapy, most commonly for back pain, other musculoskeletal pain, or for psychosocial problems or stress [6,7]. Chiropractic was the most popular method mentioned, followed by massage therapy, herbal therapies or supplements, and acupuncture. Research findings show that greater than 50% of CAM users seek such care because they believe that CAM combined with conventional medical care would be helpful, suggesting that CAM does not substitute for conventional care. Three in 10 CAM users believe that conventional care alone would not be effective for their condition [3] and greater than six in 10 of CAM patients do not tell their physicians about their use of CAM [8,9] and users of both CAM and conventional care for back or neck problems perceived CAM as more helpful than conventional care [9].

Although previous studies have compared chiropractic and medical patients, all have focused on care for specific conditions and in select geographic areas [10-15]. Multinational comparisons of all care seekers are of increasing relevance. Canada and the United States are countries with many similarities yet key differences in health-care policy and delivery. For example, the majority of medical doctors in Canada are in primary care, which is fully covered, whereas in the U.S., specialists outnumber primary care doctors, and lack of insurance is widespread [16]. Greater primary-care-to-specialist ratios have been associated with better population health indices [17].

The objective of the current study is to compare and contrast the socioeconomic and demographic, clinical and behavioral characteristics, and health-care perceptions of persons seeking care from chiropractors and general practitioners in the United States and Canada. The specific aims are to (1) estimate general practitioner and chiropractic utilization rates in Canada and the U.S., (2) identify factors associated with seeking (a) any chiropractic care vs. general practitioner care alone, and (b) chiropractic care alone vs. general practitioner and chiropractic care in Canada and the U.S., and (3) identify between-country differences affecting the use of chiropractors vs. general practitioners.

## Methods

### Data source, subject selection, and target population

Public-use data from the Joint Canada/United States Survey of Health (JCUSH), 2002–03, were accessed and analyzed [18]. The JCUSH was a one-time stratified random sample telephone survey of non-institutionalized adult (aged 18 or greater) residents of Canada (N = 3505) and the U.S. (N = 5183) conducted between November 2002 and June 2003. Households were selected via a random digit dialing (RDD) process, and all interviews were conducted from the regional offices of Statistics Canada using the Computer-Assisted Telephone Interviewing (CATI) method [19]. The interview was administered in either French or English for Canadian respondents, and in either Spanish or English for U.S. respondents. The survey response rates were 66% and 50% in Canada and the U.S., respectively [19]. The target population was persons 18 years old or older residing in private dwellings with land-line telephones in Canada and the U.S., excluding persons living in the territories of Canada or the U.S. The target population sizes in Canada and the U.S. were 24,046,837 and 206,417,185, respectively [20,21].

### Chiropractic and general practitioner utilization

Respondents were queried about the number of visits to or contacts with various types of health professionals, including family doctors or general practitioners and chiropractors. Numbers of provider-specific visits per year were capped at 31 or more. Respondents within each country were categorized according to their type of reported health-care utilization in the past 12 months: any chiropractic care (DC), family doctor or general practitioner care only (GP), both DC and GP care, and DC without GP care.

### Socioeconomic and demographic factors

Socioeconomic and demographic variables were age (18–44, 45–64, >64), sex, race/ethnicity (white only, other/multiple), marital status (married/with partner, widowed, separated/divorced, single), highest level of school completed or highest degree received (no high school degree, high school degree, some college, 4-year college degree), main source of income (employment vs. other), and amount of household income (adjusted for household size and placed in quintiles).

### Health status and reported chronic conditions

Current general health status was assessed with several measures, including a 5-point measure of self-rated general health (excellent, very good, good, fair, poor), and presence of one or more chronic conditions (e.g., conditions that had lasted or were expected to last 6 months or more and had been diagnosed by a doctor or other health professional). Conditions included asthma, osteoarthritis, rheumatoid arthritis, hypertension, emphysema or chronic obstructive pulmonary disease, diabetes, heart disease, coronary heart disease, angina pectoris, and heart attack history. The reported chronic conditions were summed to create a chronic condition index ranging from 0 (no reported conditions) to 10.

Mental health status, specifically depression, was assessed with a subset of questions from the Composite International Diagnostic Interview (CIDI) [22], which covers depressive disorder symptoms itemized in the Diagnostic and Statistical Manual of Mental Disorders (DSM-III-R) and produces diagnoses according to the Diagnostic Criteria for the Research of the ICD-10 [23]. Responses were transformed into a probability estimate of a diagnosis of major depressive episode (MDE) in the past 12 months. Respondents with estimates reflecting a 90% or greater certainty of a positive diagnosis (0.9 or greater) were classified as having had major depressive episodes in the past year [22-24]. Visits to mental health professionals were also tabulated.

Health-related quality of life was measured with the well validated Health Utility Index (HUI) [25]. The HUI includes components related to vision, hearing, speech, mobility, dexterity, emotions, cognition, and pain and discomfort [25].

### Activity restrictions and lifestyle factors affecting health

The JCUSH included queries on restriction of activities (sometimes, often, never) due to one or more chronic health conditions, and activity limitations due to pain (no pain, pain but no activity limitations, pain prevents a few activities, pain prevents some activities, pain prevents most activities). Respondents were asked about the specific conditions or health problems responsible for any difficulties with performing activities of daily living, with back or neck problems among the specific response options. Respondents reporting pain rated the usual intensity of their pain or discomfort as being mild, moderate, or severe.

Respondents were asked about their smoking status, height and weight, and physical activity levels. Current smokers were those individuals who reported having smoked at least one whole cigarette and at the time of the survey smoked cigarettes every day. Each person's body mass index (BMI) was computed by dividing weight in kilograms by the square of height in meters. The World Health Organization's categories for classifying persons according to BMI are used [26]: underweight (<18.5), normal weight (18.5 – <25), overweight (25 – <30), and obese (> = 30). Activity-specific metabolic equivalent task (MET) scores [27,28] and responses to questions about the frequency and duration of participation in leisure time physical activities in the past 3 months were used to compute total daily energy expenditure [29], which were then used to classify persons as being physically active (> = 3), moderately active (1.5 – <3), or inactive (<1.5). Respondents were also classified according to their frequency of physical activity lasting more than 15 minutes in the past 3 months (regular [> = 12], occasional [4 – <12], infrequent [<4]) [30].

### Health-care utilization, perceived unmet needs, and satisfaction with care

The JCUSH included several items on hospitalizations and the use of and visit frequency to medical doctors and other health care professionals in the past 12 months; prescription medication use in the past month and number of medications taken in the past 2 days; and in the U.S., health insurance status during the past 12 months. Respondents were also asked if they needed a health-care service in the past 12 months but didn't receive it because of lack of access, cost, or other reason (unmet health care need); and about their satisfaction with the overall quality of health care in the past 12 months, with physician care during their most recent visit (excellent, good, fair, poor), and the way health care services and physician care were provided (very satisfied, somewhat satisfied, neither satisfied nor dissatisfied, somewhat dissatisfied, very dissatisfied).

### Statistical methods

Distributions and percentages weighted to reflect the complex survey design and nonresponse were produced according to each variable, stratified by pattern of health-care use and country. The survey weight corresponds to the number of persons represented by the respondent for the target population [19]. Post-stratification using age, sex, region (Canada only), and race/ethnicity (U.S. only) was performed to ensure that the final weights sum to the population estimates, based on Canada's 1996 Census of Population [20] and the United States' October 2002 Current Population Survey [21]. SAS was used for data management and preliminary statistical analysis [31]. Because of the need to account for the complex survey design when estimating variances and confidence intervals, SUDAAN was used in modeling of associations and variance estimation [32]. SUDAAN uses the Taylor series method for estimation of variances.

Logistic regression modeling was employed to estimate crude and adjusted associations (odds ratios and 95% confidence intervals) of each factor on care seeking from chiropractors vs. general practitioners. Specifically, two sets of crude and adjusted models were run: one to estimate associations of potential predictors with any chiropractic care vs. general practitioner care only, and another to estimate associations of potential predictors with chiropractic care only vs. both chiropractic and general practitioner care. Sets of one or more binary (dummy) variables were used to model the effects of all predictors, with the exceptions of the health utility, chronic condition, and prescription medication indices, which were modeled as continuous variables, and age, which was modeled as both a categorical and a continuous predictor. Potential between-country differences in effect estimates were evaluated by the inclusion of interaction terms in additional sets of multivariable models.

Potential confounders were identified a priori as those measured variables thought to be predictors of type of health-care use and possibly associated with one or more of the selected variables of interest. Because of inherent multidirectional relations (e.g., variables acting as both causes and consequences of certain predictors and/or health-care use) and lack of longitudinal data, two sets of separate multivariable logistic regression models were built to estimate adjusted associations. In addition to the selected variable (e.g., potential predictor), one set of models included only age, sex, and race/ethnicity. The second set included these variables plus education, main source of income, health-related quality of life (HUI), and health insurance status (U.S. only).

To assess the sensitivity of estimates to method of health-status measurement and to avoid multicollinearity, alternative models replaced the HUI with self-rated general health, chronic condition index score, and activity limitations due to pain. Because replacing the HUI with each of these variables, alone and in combination, did not materially change the odds ratios, these effect estimates are not presented.

### Human subjects

The JCUSH design, questionnaires, and informed consent, interview and all other survey-related protocols were reviewed and approved by the Institutional Review Boards of Statistics Canada and the United States National Center for Health Statistics [19]. Because all direct identifiers, plus any characteristics that could possibly lead to the identity of any individual respondent, are removed from the public-use data files, which can only be used for statistical research and data analysis, and cannot be linked to any other individually identifiable data, the project qualified for exemption from coverage by the Human Subjects regulations.

## Results

### Frequency of chiropractic and general practitioner care in Canada and the U.S

Table 1 shows the sociodemographics, health characteristics, and health-care utilization of (1) all respondents and (2) respondents reporting any chiropractic care, by country. In both the U.S. and Canada, about 12% of adults seek chiropractic care annually, nearly 80% seek GP care, two-thirds seek GP care and no chiropractic care, about 10% visit both chiropractors and general practitioners, and fewer than 2% seek only chiropractic care. Estimated DC visit rates in the U.S. and Canada are 0.94 and 1.14 per 100 person-years, respectively. General practitioner visit rates are 2.84 and 3.31 per 100 person-years in the U.S. and Canada, respectively.

### DC vs. GP patient comparisons

#### Socioeconomic and demographic factors

Table 2 shows the distributions of socioeconomic and demographic characteristics of DC and GP patients, by country. Compared with GP only patients in both the U.S. and Canada, GP patients who also saw DCs are younger and more likely to be white, female, married, college educated, in the highest income quintile, and to report employment as their main income source. Compared with persons who sought care from both GPs and DCs, DC only patients in both countries tend to be younger, male, single, less educated, to have lower incomes, and to report employment as their main income source.

#### Health status and reported chronic conditions

Table 3 gives the distributions of general and mental health status, and frequencies of chronic conditions reported by DC and GP patients, by country. Compared with GP only patients, GP patients who also saw DCs in the U.S. and Canada are more likely to be in very good or excellent self-reported health and to have fewer chronic health conditions, but more likely to report histories of asthma and/or arthritis. DC patients in the U.S. are more likely to have had a depressive episode and mental health visits in the past year, whereas Canadian DC patients were more likely than their GP counterparts to have mental health visits and to be happy and interested in life. Compared with persons who sought care from both GPs and DCs, DC only patients in both countries tend to have fewer chronic health conditions.

#### Activity restrictions, weight, smoking, and physical activity

Table 4 compares DC and GP patients on variables related to activity restrictions, body weight, smoking, and physical activity, by country. Compared with GP only patients, GP patients who also saw DCs in the U.S. and Canada are more likely to have had activity-limiting back or neck problems in the past year. Compared with persons who sought care from both GPs and DCs, DC only patients tend to have less activity-limiting back or neck pain, and fewer activity restrictions. Most patients in the U.S., regardless of pattern of care seeking, are overweight or obese, never smokers, and physically inactive. Canadian patients are somewhat less likely than U.S. patients to be overweight or obese, never smokers, and physically inactive.

#### Health-care use and perceptions

Table 5 compares DC and GP patients on health-care and prescription-medication use, satisfaction with care and with providers, and unmet health-care needs, by country. Overall visit rates vary dramatically according to whether persons seek care from a DC or a GP, though GP visit rates were similar among GP patients who sought and did not seek chiropractic care, suggesting that chiropractic visits did not substitute for GP visits among these patients. However, compared with GP patients who did not seek chiropractic care, GP patients with chiropractic visits had on average almost 1.5 more visits to other health-care providers in both Canada and the U.S. Conversely, chiropractic patients with no GP visits had fewer visits, on average, to other health-care providers.

Compared with GP only patients, Canadian GP patients who also saw DCs reported greater dissatisfaction with their overall health and physician care. In the U.S., patients who saw GPs only and those who saw both GPs and DCs were comparable to each other in these and other health-care respects, including reported unmet health-care needs, which are greater overall in the U.S. than in Canada. Compared with persons who sought care from both GPs and DCs, DC only patients in both countries were less likely to have been hospitalized in the past year, less likely to use prescription drugs, less likely to have a regular doctor, more dissatisfied with their overall health care, and in the U.S., more likely uninsured.

### Factors associated with chiropractic vs. general practitioner care in Canada and the U.S

Tables 6 and 7 show the estimated crude and adjusted associations of socioeconomic and demographic, quality of life, and health-related factors on seeking in the past 12 months (1) any chiropractic care vs. GP only care and (2) chiropractic care only vs. both GP and DC care among U.S. (Table 6) and Canadian (Table 7) respondents. The age-sex-race/ethnicity-adjusted estimates (Model 1) were mostly unaffected by the additional control of education, main source of income, health-related quality of life, and (in the U.S.) health insurance status (Model 2).

Factors associated with seeking chiropractic care vs. GP care alone in the U.S. are being less than 65 years old and white; having arthritis, bodily pain, and activity limitations due to back or neck pain; being obese, and lacking a regular doctor. Canadian chiropractic care seekers, compared with GP patients, tend to be under 65, white, and college educated; report higher incomes and greater happiness with life; have arthritis, bodily pain, and activity limitations due to back or neck pain; and perceive the quality of physician care to be fair or poor, and to not be satisfied with it.

Adjusted effect estimates (odds ratios) of factors on seeking any DC care vs. GP only care that vary appreciably by country (U.S. vs. Canada) are having a regular doctor (0.59 vs. 0.95, *P *= 0.08), perceiving health care quality as good (0.93 vs. 1.24, *P *= 0.09) or fair or poor (0.95 vs. 1.36, *P *= 0.15) vs. excellent, perceiving quality of MD care as good (0.94 vs. 1.23, *P *= 0.14) or fair or poor (0.71 vs. 2.25, *P *= 0.0008) vs. excellent, and being somewhat satisfied (0.77 vs. 1.17, *P *= 0.04) or not satisfied (0.72 vs. 1.65, *P *= 0.02) vs. very satisfied with MD care.

Compared with persons who sought care from both GPs and DCs in the U.S., DC only patients are relatively younger, with no or few chronic conditions, on no or few prescription medications, to have no regular doctor, no health insurance, and to express dissatisfaction with the quality of health care. Factors associated with DC only vs. DC and GP care among Canadian respondents are age less than 45, being male, and lacking a college degree; having better self-rated health, no or few chronic conditions, and no bodily pain or activity limitations due to back or neck pain; being a current smoker, not obese, and infrequently physically active; taking no or few prescription drugs and lacking a regular doctor.

Adjusted effect estimates (odds ratios) of factors on seeking DC care only vs. GP and DC care that vary appreciably by country (U.S. vs. Canada) are 10-year age increment (0.90 vs. 0.61, *P *= 0.10), male sex (1.34 vs. 3.63, *P *= 0.04), fair or poor vs. excellent self-rated health (0.69 vs. 0.09, *P *= 0.11), depression in the past year (1.08 vs. 0.39, *P *= 0.15), pain-related activity limitations (1.12 vs. 0.30, *P *= 0.06), disabling back or neck problems (1.04 vs. 0.19, *P *= 0.07), past (1.32 vs.0.22, *P *= 0.01) and current (0.68 vs. 2.13, *P *= 0.03) smoking, being overweight (1.64 vs. 0.55, *P *= 0.15), infrequent physical activity (1.11 vs. 2.42, *P *= 0.13), and having a regular doctor (0.09 vs. 0.25, *P *= 0.08).

## Discussion

This is the first study, to our knowledge, using nationally-representative samples from more than one country and the same survey methods and measures to (1) document the similarities and differences of persons seeking care from general practitioners and those seeking care from chiropractors, alone or in conjunction with GP care, and (2) provide mutually comparable estimates of chiropractic and GP visit rates. The only other study to estimate chiropractic visit rates in the U.S. and Canada found the visit rates to be 101.2 and 140.9 per 100 person years, respectively [33]. These estimates are derived from 5 sites in the U.S. and from Toronto, Ontario, Canada, and thus may not be entirely representative of the national populations. Because the JCUHS capped the annual number of provider-specific visits at 31 or more, our lower estimates of 0.94 and 1.14 per 100 person-years, respectively, are conservative and likely somewhat less than the true chiropractic visit rates in each country, however.

Extrapolating to the adult populations of the U.S. and Canada, approximately 23.5 million adults in the U.S. and 3.1 million adults in Canada had at least one chiropractic visit in the past year. Although the vast majority of chiropractic patients also sought care from primary-care medical doctors, about 3.5 million chiropractic patients in the U.S. and almost 400,000 in Canada did not seek care from general or family practitioners, suggesting that chiropractors may be delivering or in the position to deliver primary health care to an appreciable number of people, and indicating the need for chiropractors to be knowledgeable and cognizant of problems that may require medical referral. More than 50% of these patients in the U.S. and 35% in Canada reported not having a regular doctor. Large proportions of chiropractic patients have chronic conditions, recent episodes of depression, and reported use of prescription medications, underscoring the importance for chiropractors in taking thorough health and medication inventories, querying about possible drug side effects and interactions, offering drug-free alternatives to their patients, and integrating chiropractic with conventional medical care [34]. Given that persons seeking chiropractic care are just as likely as others to smoke and be overweight or obese and sedentary, the role for chiropractic in encouraging health promotion and disease prevention activities also cannot be underestimated [35].

Differences in the characteristics of persons seeking care from chiropractors alone vs. care from both medical and chiropractic providers are notable. For example, persons seeking care from chiropractors are more likely than those seeking primary medical care to have activity limitations due to back or neck pain. However, persons seeking *only *chiropractic care in Canada are less likely to have activity-limiting back or neck pain, more likely uninsured in the U.S., and in both countries, much less likely to have chronic conditions and to have a regular doctor. These differences indicate that the types and patterns of care may vary between these two populations. Persons seeking both medical and chiropractic care may use chiropractic for musculoskeletal or neuromusculoskeletal problems, whereas persons who seek chiropractic care alone may use it more for wellness or preventive care, or, given lack of health insurance and lack of a regular doctor, for primary care as well. These distinctions have important implications for chiropractic health-care delivery models, which may necessarily vary and evolve within and across populations according to provider access, inter-professional cooperation and referral relationships, quality of available medical care, health insurance and economic resources, and provider and patient attitudes and beliefs, among others [15,36-43].

These findings suggest that the relative integration of chiropractic with medical care, and chiropractic's role in primary health-care delivery (vs. back and neck pain care), in North America and around the globe, are likely dependent on social, cultural, and environmental factors, individual and community resources, and personal preferences and predilections. Recent research has uncovered vast deficiencies in the delivery of primary care in several countries, but most notably in the U.S. [16]. Failure to (1) provide patient-centered care, (2) emphasize preventive care, (3) be aware of patients' health concerns, and (4) effectively manage chronic conditions are a few of the shortcomings identified. These deficiencies may be reflected in patients' perceptions of health-care quality and satisfaction, driving primary-care medical patients toward chiropractic. Given chiropractic's emphasis on disease prevention, health maintenance, and the patient-doctor relationship [5,38,40,44], chiropractic may have a role in improving the effectiveness of and access to primary care domestically and internationally [43], and perhaps help to reduce health inequalities within and between countries [45]. Challenges abound, however, in the U.S. and around the world (40, 44). For example, according to the World Federation of Chiropractic's October 2004 survey of almost 3700 respondents worldwide [44], perceptions of the general public and medical doctors toward chiropractic vis-a-vis primary health care vs. management of back and neck pain differ markedly from how the chiropractic profession believes the public and medical doctors should perceive chiropractic. Furthermore, surveys of chiropractors and chiropractic patients from several countries around the world show that the vast majority of patients seek care for relief from back and neck pain and other neuromusculoskeletal complaints [46-52]. Although some between-country differences in patients have been observed, the use of consistent measures and methods across studies would be necessary to better estimate and interpret observed differences in patient populations [49].

The only other study using a representative sample of the U.S. population to compare users of medical care and chiropractic care identified several factors associated with the type of back care among those who sought health care for their back problem [13]. Specifically, adults with disabling comorbidities and back-related restricted-activity days were relatively less likely to use chiropractic care than primary medical care. Those who were male, white, high-school educated, single, employed, living in the West, and reporting more than nine doctor visits during the previous 12 months, a back condition of more than five years' duration, and no back-related disability were relatively more likely to use chiropractic care compared with primary medical care. Chiropractic patients also had better self-perceived general health status, fewer bed and restricted-activity days, and fewer comorbid conditions, compared with patients of other providers [13,14]. Additional studies of health-care use for back pain in the U.S. have found that residents in the West [10], whites [10,11,46], males [11], high-school graduates [11,52], chronic back-pain sufferers [53], and persons with good-to-excellent self-reported health [12], adequate health insurance [12], and with less severe pain [12] are more likely to visit a chiropractor.

One Canadian study, a population-based cross-sectional survey of the Saskatchewan population 20–69 years old, found that persons with back or neck pain were more likely to consult a chiropractor alone rather than a medical doctor if they were younger, male, living in urban areas, not in the lower socioeconomic categories, and without arthritis, and with fewer comorbidities, less severe pain, and better physical and social functioning and higher scores on other health-related quality of life measures [15]. These results are generally not inconsistent with our findings among Canadian respondents, and compatible with a generally healthier segment of the population with back or neck pain having self selected chiropractic care. However, a study comparing chiropractic back pain patients with medical patients based primarily in the U.S. found that chiropractic patients had much worse mental health scores [36]. This finding is compatible with our results showing a higher prevalence of depression among chiropractic care seekers in the U.S., and a higher prevalence of mental health visits among chiropractic care seekers in Canada. Given that persons with back pain often have comorbid depression, as a cause, consequence or associated condition occurring with back pain through shared neural, immune, psychological, or other pathways [54,55], a not unsurprising finding. Asthma has also been shown to be associated with back pain and depression, possibly explaining the somewhat higher prevalence of asthmatics among chiropractic patients [56].

Major strengths of the study are the population-based design covering two countries and several nationalities, survey administration in three languages using a standard approach across countries, the CATI method of questionnaire administration, the comprehensive set of previously validated measures encompassing multiple dimensions (e.g., health status, chronic conditions, activity restrictions, socioeconomic and demographic information, lifestyle factors, use of and access to health-care services, perceptions of health-care quality and satisfaction), use of several sets of multivariable models to control for potential confounding and to assess sensitivity of estimates, and methods allowing findings to be generalized to the U.S. and Canada. Quality assurance measures, including use of skilled interviewers, observations of interviewers, use of procedures to ensure that data errors were minimized, and coding and edit quality checks to verify processing logic were employed to reduce non-sampling errors [19].

Our findings should be considered in light of several limitations. Despite taking into account non-response in the analyses, the relatively low response rates may have introduced selection bias because of possible differences between respondents and nonrespondents. We do not have any data on which to compare these two groups and estimate the magnitude of the bias, if any. All data were self reported and no attempts were made to verify the accuracy of the reports, either through direct observation or via independent sources. Bias resulting from inaccurate recall or dishonesty may have occurred. Residual confounding is another source of possible bias. We used multivariable modeling to control for the effects of several variables; however, confounding cannot be ruled out. For example, population density and geographic area, which have been shown previously to be associated with care seeking [10,13,15,33], are not included in the survey and thus could not be considered analytically as either predictors of use or confounders of other exposure effects. Because the study is cross sectional in nature and many of the variables are time dependent, the temporal relations between several of the potential predictors and pattern of health-care use cannot be determined. The study also does not allow for inferences regarding care-seeking for specific conditions, or the effects of care seeking on clinical, cost, and other outcomes. Finally, estimates at the state and provincial levels are not possible, and because of small numbers of persons seeking care from chiropractors alone, some parameters were not estimable or estimated imprecisely.

## Conclusion

Chiropractic and general practitioner patients are quite dissimilar within both Canada and the U.S., and notable between-country differences in GP and DC patients are apparent as well. Such individual and geographic variations have broad and potentially far-reaching implications for patients, providers, policymakers, and researchers. Our findings should be considered when interpreting observational studies of chiropractic and medical care utilization and outcomes, and would be useful in the design of longitudinal studies to test specific hypotheses regarding individual and societal-level predictors and consequences of (a) chiropractic vs. primary medical care and (b) the integration of chiropractic with medical care in North America and elsewhere.

## Competing interests

The author(s) declare that they have no competing interests.

## Authors' contributions

ELH conceived of the study, developed the objectives, reviewed the literature, supervised the analysis, and drafted the manuscript. L-MC performed the statistical analysis and helped to draft part of the manuscript. Both authors read and approved the final manuscript.

## Pre-publication history

The pre-publication history for this paper can be accessed here:



## Figures and Tables

**Table 1:** Frequency distributions (weighted percentages unless indicated otherwise) of socioeconomic, demographic, and health characteristics of respondents, and their health-care utilization, by country: Joint Canada/US Survey of Health, 2002–03*

		United States		Canada	
Variable	Category	Any DC care (n = 595)	Total pop. (N = 5183)	Any DC care (n = 448)	Total pop. (N = 3505)
*Socioeconomic and demographic factors:*					
Age (Years)	18–44	50.3	52.3	56.4	52.3
	45–64	37.2	31.7	32.8	32.1
	≥ 65	12.5	16.0	10.8	15.6
	Mean (SE)	45.2 (0.7)	45.4 (0.3)	43.4 (0.8)	45.1 (0.3)
	Median	44	43	42	43
Sex	Male	43.4	48.0	47.0	49.1
	Female	56.6	52.0	53.0	50.9
Race/ethnicity	White only	80.2	69.3	86.8	81.0
	Other/multiple	16.7	26.4	13.0	17.7
Education	No high school degree or GED	8.4	11.3	15.1	19.4
	High school degree or GED	35.8	35.8	32.4	30.6
	Some college	14.1	35.3	20.2	21.2
	4-year college degree or certificate	39.0	35.3	31.2	27.3
Marital status	Married or living with partner	65.9	61.3	69.6	64.7
	Widowed	4.6	6.1	3.9	5.7
	Separated or divorced	11.4	10.4	7.8	7.7
	Single	15.3	18.5	18.4	20.9
Household income (adjusted for household size)	Lowest quintile	11.3	15.3	11.5	17.0
	Lower middle	18.0	16.4	18.9	17.5
	Middle	15.4	14.5	16.6	16.1
	Upper middle	18.3	15.2	20.2	17.6
	Highest	18.2	14.8	21.6	16.0
Main income source	Employment	77.3	72.4	79.7	75.0
*Health status and chronic conditions:*					
Chronic conditions (lifetime history)	Asthma	13.1	11.4	12.3	10.4
	Arthritis	23.2	18.6	20.3	16.8
	Hypertension	19.9	22.7	13.0	18.3
	Emphysema or COPD	1.8	2.1	1.2	1.3
	Diabetes	5.1	6.7	3.9	4.7
	Heart disease	4.7	6.0	4.5	5.1
	Coronary heart disease	2.1	2.4	1.4	1.8
	Angina	1.5	2.9	2.1	3.7
	1 or more conditions	38.4	36.8	34.2	33.4
Depression	Episode in past 12 months	11.2	8.5	9.0	8.0
Mental health visit	Visit in past 12 months	14.8	10.5	14.5	10.1
Emotional problems	Happy and interested in life	77.7	77.9	84.8	80.8
Health Utility Index	Mean (SE)	0.87 (0.01)	0.87 (<0.01)	0.87 (0.01)	0.88 (<0.01)
Self-rated health	Excellent	26.1	26.4	23.5	24.0
	Very good	36.9	32.5	39.1	36.4
	Good	24.9	26.5	25.3	28.0
	Fair or poor	12.1	14.6	12.2	11.5
*Activity restrictions and lifestyle factors:*					
Pain/activity limits	No pain or discomfort	75.2	80.6	75.3	82.0
Activity restrictions	Often	9.9	10.1	10.0	9.6
	Sometimes	24.4	18.6	25.4	19.8
	Never	64.6	69.9	63.9	69.6
Back or neck problems	Cause of activity limitations	16.3	9.1	20.1	10.4
Smoking status	Current	19.7	22.3	23.6	24.8
	Past	28.4	23.5	31.5	28.7
	Never	51.5	53.7	44.7	46.2
Body weight	Under/normal weight (BMI <25)	40.4	43.5	47.6	48.8
	Overweight (BMI 25 – <30)	32.9	32.5	35.2	32.7
	Obese (BMI > = 30)	22.9	19.8	14.8	14.8
Physical activity (past 3 months)	Active	22.0	21.1	27.0	26.4
	Moderate	20.7	20.2	25.2	25.5
	Inactive	55.2	55.9	46.9	47.0
Activity frequency (past 3 months)	Regular	56.2	55.0	67.1	65.8
	Occasional	18.3	16.9	18.4	16.4
	Infrequent	23.5	25.2	13.7	16.7
*Health-care use and perceptions:*					
Hospitalization	1 or more in past 12 months	10.3	10.2	7.7	9.1
No. of prescriptions	1 or more in past month	66.6	57.5	57.8	55.2
No. of different prescription medications	<3 in past 2 days	47.3	37.5	44.4	39.9
	3–5 in past 2 days	15.6	14.2	9.8	11.7
	6+ in past 2 days	3.7	5.6	3.0	3.3
Health insurance	Insured in past 12 months	89.2	86.9	NA	NA7
Unmet health-care needs	Any in past 12 months	12.5	13.1	11.6	10.7
Regular doctor	Yes in past 12 months	84.3	79.5	89.2	84.7
Health care quality (past 12 months)	Excellent	41.6	38.2	33.6	36.5
	Good	44.4	42.5	48.1	43.3
	Fair or poor	11.1	10.5	16.7	13.9
Health care satisfaction (past 12 months)	Very satisfied	50.8	48.5	41.2	40.8
	Somewhat satisfied	35.9	33.3	40.7	40.4
	Less than satisfied	10.3	9.1	16.1	12.1
Quality of MD care^† ^(past 12 months)	Excellent	59.6	56.6	52.3	58.3
	Good	32.6	32.6	36.0	33.1
	Fair or poor	5.2	6.8	11.1	6.9
Satisfaction of MD care^† ^(past 12 months)	Very satisfied	69.2	65.2	61.0	64.8
	Somewhat satisfied	23.2	25.7	29.2	27.0
	Less than satisfied	4.2	4.8	8.9	6.1
Health-care use^‡ ^(past 12 months)	Any chiropractic care	100	11.4	100	12.9
	GP care only	0	67.2	0	68.8
	Both GP and DC care	85.3	9.7	87.7	11.3
	Chiropractic care only	14.7	1.7	13.3	1.6
	No GP or DC care	0	20.7	0	17.5
Chiropractic visits (past 12 months)	0	0	88.5	0	86.8
	1	20.9	2.4	14.7	1.9
	2–5	34.9	4.0	34.1	4.4
	6–10	15.8	1.8	20.4	2.6
	11–20	18.0	2.1	19.5	2.5
	21–30	4.6	0.5	6.8	0.9
	31+	5.8	0.7	4.5	0.6
	Mean, median	8.22, 4	0.94, 0	8.80, 5	1.14, 0
General practitioner or family doctor visits (past 12 months)	0	14.7	22.5	12.3	19.1
	1	22.4	22.8	19.8	21.8
	2–5	50.0	41.8	49.7	42.8
	6–10	8.2	7.4	9.0	7.9
	11–20	2.7	3.9	8.0	6.6
	21–30	1.1	0.7	1.0	0.7
	31+	0.9	0.5	0.2	0.5
	Mean, median	3.18, 2	2.84, 2	3.69, 2	3.31, 2
All health-care visits (past 12 months)	0	0	6.9	0	5.2
	1	0.7	7.3	0.9	7.9
	2–5	14.6	38.6	11.4	39.0
	6–10	24.1	22.1	22.4	22.3
	11–20	28.7	15.3	32.3	15.5
	21–30	14.7	4.9	17.6	5.7
	31+	17.2	4.9	15.5	4.5
	Mean, median	17.11, 14	8.69, 5	17.97, 15	8.66, 5

* Weighted percentages may not sum to 100 because of missing data or exclusion of certain categories; percentages weighted to account for age, sex, region (Canada only), and race/ethnicity (U.S. only); 595 and 5183 represent 23,513,576 and 206,417,185 adults, respectively, in the U.S.; 448 and 3505 represent 3,105,017 and 24,046,837 adults, respectively, in Canada.

^† ^Most recent visit among respondents reporting at least one visit to a physician in the past 12 months.

^‡ ^Weighted percentages sum to greater than 100 because categories are not mutually exclusive.

DC = Doctor of Chiropractic; SE = standard error; COPD = chronic obstructive pulmonary disease; BMI = body mass index; NA = not applicable; GP = general practitioner or family doctor; MD = medical doctor.

**Table 2:** Frequency distributions (weighted percentages unless indicated otherwise) of socioeconomic and demographic characteristics of DC and GP patients, by country and pattern of health-care use in the past 12 months: Joint Canada/US Survey of Health, 2002–03*

		United States			Canada		
Variable	Category	GP only (n = 3565)	GP+DC (n = 511)	DC only (n = 84)	GP only (n = 2434)	GP+DC (n = 397)	DC only (n = 51)
Age (Years)	18–44	47.8	47.6	65.7	48.5	53.0	81.1
	45–64	33.0	38.8	28.3	32.8	35.2	15.6
	≥ 65	19.2	13.6	6.1	18.7	11.8	3.3
	Mean (SE)	47.2 (0.3)	46.1 (0.8)	40.3 (1.4)	46.5 (0.4)	44.6 (0.8)	34.4 (2.0)
	Median	45	45	38	45	43	33
Sex	Male	45.1	42.2	50.5	45.5	42.7	77.2
	Female	54.9	57.8	49.5	54.5	57.3	22.8
Race/ethnicity	White only	68.9	81.4	73.3	80.1	86.9	85.5
	Other/multiple	26.7	15.4	24.1	18.4	12.8	14.5
Education	No H.S. degree	10.8	8.0	10.4	20.7	13.9	23.3
	High school degree	35.4	35.1	39.6	29.2	31.5	38.4
	Some college	13.9	13.0	20.0	21.7	20.0	22.1
	4-year college degree 35.6	41.1	27.3	27.0	33.3	16.1	
Marital status	Married/with partner	61.8	66.2	64.0	64.9	70.9	60.6
	Widowed	7.1	4.6	4.1	6.7	4.5	0.0
	Separated/divorced	10.3	11.5	10.8	7.5	8.5	2.8
	Single	16.9	14.8	18.5	19.7	15.9	36.6
Household income (adjusted for household size)	Lowest quintile	14.8	10.5	15.5	18.2	11.5	11.6
	Lower middle	16.3	18.4	15.8	17.0	18.2	24.2
	Middle	15.1	15.7	13.7	16.6	16.2	19.5
	Upper middle	14.8	18.7	15.9	16.9	20.6	16.9
	Highest	14.9	18.5	16.8	15.2	22.8	13.1
Main income source	Employment	69.6	76.5	82.1	71.6	79.4	82.0

* Weighted percentages may not sum to 100 because of missing data or exclusion of certain categories; percentages weighted to account for age, sex, region (Canada only), and race/ethnicity (U.S. only); 3565, 511, and 84 represent 138,802,519, 20,060,412, and 3,453,164 adults, respectively, in the U.S.; 2434, 397, and 51 represent 16,555,719, 2,723,870, and 381,147 adults, respectively, in Canada.

GP = general practitioner or family doctor; DC = Doctor of Chiropractic; SE = standard error.

**Table 3:** Frequency distributions (weighted percentages unless indicated otherwise) of health status variables and chronic conditions reported by DC and GP patients, by country and pattern of health-care use in the past 12 months: Joint Canada/US Survey of Health, 2002–03*

		United States			Canada		
Variable	Category	GP only (n = 3565)	GP+DC (n = 511)	DC only (n = 84)	GP only (n = 2434)	GP+DC (n = 397)	DC only (n = 51)
Health Utility Index	Mean (SE)	0.86 (<0.01)	0.87 (0.01)	0.87 (0.02)	0.88 (0.01)	0.87 (0.01)	0.92 (0.02)
Self-rated health	Excellent	23.4	25.3	30.7	20.8	21.5	38.0
	Very good	32.7	38.5	27.7	36.9	39.6	35.4
	Good	27.3	23.4	34.0	29.3	25.3	24.7
	Fair or poor	16.6	12.8	7.6	12.9	13.6	2.0
Depression	Episode, past year	8.7	11.2	11.6	8.5	9.8	3.5
Mental health visit	Visit, past year	11.8	16.1	7.5	11.1	16.5	0.0
Emotional problems	Happy in life	78.0	77.4	79.0	80.3	84.9	84.0
Chronic conditions (lifetime history)	Asthma	12.0	14.8	3.3	10.8	12.5	10.4
	Arthritis	21.3	24.2	17.8	19.1	23.2	0.0
	Hypertension	27.4	22.7	3.5	22.0	14.4	2.5
	Emphysema or COPD 2.6	2.0	0.7	1.6	1.4	0.0	
	Diabetes	8.6	6.0	0.0	5.8	4.3	0.7
	Heart disease	7.5	5.0	3.0	6.2	5.0	1.0
	Coronary heart disease 3.1	2.3	0.6	2.3	1.4	1.0	
	Angina	3.6	1.4	2.5	4.8	2.4	0.0
	1 or more conditions	43.1	41.7	19.1	38.2	37.9	8.2

* Weighted percentages may not sum to 100 because of missing data or exclusion of certain categories; percentages weighted to account for age, sex, region (Canada only), and race/ethnicity (U.S. only); 3565, 511, and 84 represent 138,802,519, 20,060,412, and 3,453,164 adults, respectively, in the U.S.; 2434, 397, and 51 represent 16,555,719, 2,723,870, and 381,147 adults, respectively, in Canada.

GP = general practitioner or family doctor; DC = Doctor of Chiropractic; SE = standard error; COPD = chronic obstructive pulmonary disease.

**Table 4:** Frequency distributions (weighted percentages) of variables related to activity restrictions, body weight, smoking, and physical activity, by country and pattern of health-care use in the past 12 months: Joint Canada/US Survey of Health, 2002–03*

		United States			Canada		
Variable	Category	GP only (n = 3565)	GP+DC (n = 511)	DC only (n = 84)	GP only (n = 2434)	GP+DC (n = 397)	DC only (n = 51)
Pain/activity limits	No pain/discomfort	79.3	74.5	79.7	81.0	73.2	90.3
Activity restrictions	Often	11.5	10.7	5.6	11.0	10.4	6.7
	Sometimes	19.7	24.6	23.5	20.2	27.3	12.3
	Never	67.3	63.6	70.2	67.8	61.8	79.4
Back/neck problems	Activity limiting	8.9	16.9	12.8	10.0	22.2	5.5
Smoking status	Current	20.8	20.4	15.7	24.0	19.9	49.6
	Past	24.6	28.3	29.1	29.2	34.8	7.6
	Never	54.0	50.9	55.2	46.5	44.9	42.9
Body weight	Under/normal weight	42.5	40.9	37.7	48.7	46.5	55.5
	Overweight	32.4	31.4	41.6	32.3	35.4	34.1
	Obese	20.8	23.7	18.3	15.4	15.7	8.6
Physical activity (past 3 months)	Active	20.4	22.6	18.5	25.7	27.8	21.5
	Moderate	20.0	21.2	18.2	26.3	25.7	21.7
	Inactive	56.5	54.3	60.7	47.0	45.6	56.8
Activity frequency (past 3 months)	Regular	55.6	56.1	56.5	66.3	68.3	58.6
	Occasional	16.1	19.2	13.2	15.8	18.6	17.4
	Infrequent	25.2	22.8	27.7	16.8	12.2	24.0

* Weighted percentages may not sum to 100 because of missing data or exclusion of certain categories; percentages weighted to account for age, sex, region (Canada only), and race/ethnicity (U.S. only); 3565, 511, and 84 represent 138,802,519, 20,060,412, and 3,453,164 adults, respectively, in the U.S.; 2434, 397, and 51 represent 16,555,719, 2,723,870, and 381,147 adults, respectively, in Canada.

GP = general practitioner or family doctor; DC = Doctor of Chiropractic.

**Table 5:** Frequency distributions (weighted percentages) of health-care and prescription-medication use, satisfaction with care and with providers, and unmet health-care needs, by country and pattern of health-care use in the past 12 months: Joint Canada/US Survey of Health, 2002–03*

		United States			Canada		
Variable	Category	GP only (n = 3565)	GP+DC (n = 511)	DC only (n = 84)	GP only (n = 2434)	GP+DC (n = 397)	DC only (n = 51)
Hospitalization	1+ in past year	11.7	11.3	4.6	10.7	8.1	5.3
No. of prescriptions	1+ in past month	67.2	72.2	34.1	64.2	63.3	18.4
No. of different prescription medications	<3 in past 2 days	42.2	50.0	31.7	45.4	48.1	17.8
	3–5 in past 2 days	17.5	17.9	1.8	14.6	11.0	0.7
	6+ in past 2 days	7.2	4.3	0.6	4.1	3.4	0.0
Health insurance	Insured, past year	90.8	92.1	72.9	NA	NA	NA
Unmet needs	Any in past year	12.2	12.7	11.1	11.0	11.8	10.3
Regular doctor	Yes in past year	89.8	90.8	46.1	91.4	92.5	65.0
Health care quality (past 12 months)	Excellent	40.4	42.6	36.2	39.1	35.6	19.6
	Good	45.7	43.2	51.5	45.9	47.2	54.5
	Fair or poor	11.3	11.1	11.0	13.8	16.0	21.9
Health care satisfaction (past 12 months)	Very satisfied	52.0	53.2	36.8	43.4	43.1	27.2
	Somewhat satisfied	35.5	34.9	41.9	42.7	40.4	42.8
	Less than satisfied	9.7	8.6	20.0	12.3	14.7	26.0
Quality of MD care^† ^(past 12 months)	Excellent	56.7	58.4	71.5	59.7	52.5	47.8
	Good	32.7	33.9	19.1	33.0	35.9	38.2
	Fair or poor	7.1	5.0	7.8	5.8	10.9	14.0
Satisfaction of MD^† ^care (past 12 months)	Very satisfied	65.5	70.2	59.0	65.9	61.2	56.2
	Somewhat satisfied	26.2	22.1	35.0	27.0	29.2	29.9
	Less than satisfied	4.7	4.2	4.4	5.3	8.7	14.0
Chiropractic visits (past 12 months)	0	100	0	0	100	0	0
	1		20.6	23.2		13.8	21.6
	2–5		34.3	38.2		33.8	35.7
	6–10		16.8	10.2		20.9	16.9
	11–20		17.3	22.2		19.3	20.2
	21–30		5.3	0		7.3	3.2
	31+		5.8	6.2		4.8	2.5
	Mean, median		8.36, 4	7.42, 3		9.01, 5	7.29, 3
General practitioner or family doctor visits (past 12 months)	0	0	0	100	0	0	100
	1	30.0	26.3		27.9	22.6	
	2–5	53.7	58.6		52.8	56.7	
	6–10	9.6	9.7		9.8	10.2	
	11–20	5.3	3.1		8.0	9.2	
	21–30	0.8	1.3		0.8	1.2	
	31+	0.7	1.0		0.8	0.2	
	Mean, median	3.66, 2	3.73, 2		4.08, 2	4.21, 3	
All health-care visits (past 12 months)	0	0	0	0	0	0	0
	1	4.8	0	4.7	4.6	0	7.1
	2–5	43.4	10.6	38.0	45.1	8.1	34.8
	6–10	26.7	24.6	21.2	26.3	23.0	18.0
	11–20	16.4	30.4	19.3	15.7	33.3	25.5
	21–30	4.5	16.2	5.9	4.8	18.6	10.1
	31+	4.2	18.3	10.8	3.4	17.0	4.6
	Mean, median	9.02, 6	18.84, 15	11.56, 7	8.54, 6	19.04, 15	10.39, 6

* Weighted percentages may not sum to 100 because of missing data or exclusion of certain categories; percentages weighted to account for age, sex, region (Canada only), and race/ethnicity (U.S. only); 3565, 511, and 84 represent 138,802,519, 20,060,412, and 3,453,164 adults, respectively, in the U.S.; 2434, 397, and 51 represent 16,555,719, 2,723,870, and 381,147 adults, respectively, in Canada.

^†^Most recent visit among respondents reporting at least one visit to a physician in the past 12 months.

GP = general practitioner or family doctor; DC = Doctor of Chiropractic; NA = not applicable.

**Table 6:** Crude and adjusted effect estimates of socioeconomic and demographic, quality of life, and health-related factors on seeking in the past 12 months (1) any DC vs. GP care only and (2) DC only vs. both GP and DC care: Results from logistic regression analyses among U.S. respondents from the Joint Canada/US Survey of Health, 2002–03

		Any DC vs. GP Care Only (n = 4160)					DC Only vs. GP+DC Care (n = 595)				
		Adjusted effect estimates					Adjusted effect estimates				
Variable	Category	Crude OR	Model 1* OR	95% CI	Model 2^† ^OR	95% CI	Crude OR	Model 1* OR	95% CI	Model 2^† ^OR	95% CI
Age (Years)	18–44	1.00	1.00		1.00		1.00	1.00		1.00	
	45–64	1.07	1.00	(0.81, 1.24)	1.04	(0.83, 1.32)	0.53	0.57	(0.32, 1.04)	0.66	(0.36, 1.21)
	≥ 65	0.62	0.57	(0.44, 0.76)	0.73	(0.50, 1.07)	0.32	0.35	(0.16, 0.81)	0.61	(0.20, 1.84)
	Per 10 years	0.90	0.90	(0.90, 1.00)	1.00	(0.90, 1.11)	0.73	0.82	(0.67, 0.90)	0.90	(0.74, 1.11)
Sex	Female	1.00	1.00		1.00		1.00	1.00			
	Male	0.93	0.91	(0.74, 1.11)	0.93	(0.76, 1.15)	1.40	1.36	(0.80, 2.32)	1.34	(0.78, 2.32)
Race/ethnicity	White only	1.00	1.00		1.00		1.00	1.00		1.00	
	Other/multiple	0.54	0.51	(0.39, 0.68)	0.50	(0.37, 0.67)	1.74	1.57	(0.78, 3.16)	0.97	(0.48, 1.98)
Education	No H.S. degree	1.00	1.00		1.00		1.00	1.00		1.00	
	H.S. degree	1.30	1.01	(0.68, 1.50)	1.05	(0.68, 1.62)	0.87	0.84	(0.31, 2.27)	1.26	(0.38, 4.17)
	Some college	1.31	1.01	(0.65, 1.57)	1.13	(0.70, 1.80)	1.18	1.12	(0.39, 3.23)	1.83	(0.50, 6.68)
	4-year degree	1.41	1.02	(0.68, 1.51)	1.11	(0.72, 1.71)	0.51	0.49	(0.18, 1.34)	0.87	(0.25, 3.02)
Marital status	Married	1.00	1.00		1.00		1.00	1.00		1.00	
	Was married	0.86	0.97	(0.75, 1.25)	0.97	(0.74, 1.28)	0.96	1.12	(0.57, 2.20)	0.96	(0.48, 1.92)
	Single	0.85	0.81	(0.61, 1.09)	0.85	(0.63, 1.15)	1.30	1.06	(0.52, 2.17)	1.05	(0.48, 2.30)
Income (adjusted for household size)	Highest quint.	1.00	1.00		1.00		1.00	1.00		1.00	
	Upper middle	1.01	1.02	(0.73, 1.43)	1.00	(0.71, 1.40)	0.94	0.97	(0.38, 2.51)	0.93	(0.36, 2.40)
	Middle	0.84	0.91	(0.64, 1.29)	0.89	(0.62, 1.27)	0.96	0.96	(0.39, 2.37)	0.82	(0.31, 2.14)
	Lower middle	0.90	1.04	(0.73, 1.48)	1.00	(0.69, 1.46)	0.95	1.02	(0.39, 2.65)	0.51	(0.17, 1.55)
	Lowest quint.	0.62	0.79	(0.54, 1.17)	0.72	(0.46, 1.15)	1.63	1.88	(0.73, 4.83)	1.43	(0.49, 4.19)
Main income source	Employment	1.00	1.00		1.00		1.00	1.00		1.00	
	Other	0.66	0.79	(0.58, 1.07)	0.78	(0.57, 1.06)	0.52	0.69	(0.30, 1.55)	0.73	(0.26, 2.04)
HUI	Per 0.1 unit	1.02	1.00	(0.95, 1.05)	1.00	(0.95, 1.05)	1.01	0.99	(0.89, 1.12)	1.00	(0.87, 1.14)
Self-rated health	Excellent	1.00	1.00		1.00		1.00	1.00		1.00	
	Very good	1.01	1.06	(0.81, 1.37)	1.04	(0.80, 1.35)	0.59	0.63	(0.31, 1.26)	0.72	(0.35, 1.49)
	Good	0.82	0.90	(0.67, 1.19)	0.92	(0.68, 1.24)	1.20	1.30	(0.66, 2.54)	1.01	(0.48, 2.13)
	Fair or poor	0.65	0.79	(0.56, 1.13)	0.70	(0.45, 1.10)	0.49	0.62	(0.20, 1.88)	0.69	(0.20, 2.30)
Depression (past year)	1+ episodes	1.34	1.30	(0.95, 1.79)	1.31	(0.91, 1.90)	1.02	0.99	(0.43, 2.27)	1.08	(0.42, 2.75)
Mental health (past year)	1+ visits	1.29	1.19	(0.91, 1.56)	1.15	(0.84, 1.56)	0.42	0.45	(0.16, 1.23)	0.26	(0.08, 0.82)
Emotional	Happy in life	1.00	1.00		1.00		1.00	1.00		1.00	
problems	Not happy	1.03	1.02	(0.80, 1.30)	0.93	(0.69, 1.26)	0.99	1.02	(0.53, 1.98)	0.60	(0.26, 1.39)
Chronic conditions (lifetime history)	Asthma	1.11	1.12	(0.83, 1.50)	1.12	(0.83, 1.53)	0.20	0.21	(0.07, 0.58)	0.21	(0.07, 0.62)
	Arthritis	1.12	1.32	(1.02, 1.69)	1.38	(1.05, 1.81)	0.68	0.95	(0.47, 1.92)	1.12	(0.52, 2.43)
	Hypertension	0.66	0.73	(0.56, 0.94)	0.74	(0.56, 0.98)	0.12	0.13	(0.04, 0.46)	0.15	(0.04, 0.55)
	Emphysema	0.72	0.78	(0.41, 1.49)	0.80	(0.38, 1.69)	0.43	0.45	(0.05, 3.79)	0.59	(0.06, 6.13)
	Diabetes	0.57	0.71	(0.46, 1.08)	0.71	(0.45, 1.11)	^‡^	^‡^		^‡^	
	Heart disease	0.60	0.77	(0.48, 1.23)	0.83	(0.50, 1.37)	0.59	0.83	(0.26, 2.63)	1.14	(0.37, 3.49)
	CHD	0.66	0.90	(0.44, 1.85)	0.92	(0.42, 1.99)	0.25	0.30	(0.03, 2.95)	0.49	(0.06, 4.25)
	Angina	0.42	0.54	(0.28, 1.03)	0.57	(0.28, 1.14)	1.80	2.98	(0.71, 12.47)	4.09	(0.92, 18.10)
	1 or more	0.83	0.93	(0.74, 1.17)	0.94	(0.74, 1.19)	0.33	0.38	(0.20, 0.74)	0.40	(0.19, 0.82)
	Per condition	0.88	0.94	(0.86, 1.03)	0.94	(0.85, 1.05)	0.50	0.53	(0.36, 0.78)	0.56	(0.38, 0.83)
Pain/activity limits	No pain	1.00	1.00		1.00		1.00	1.00		1.00	
	Pain	1.26	1.37	(1.08, 1.72)	1.70	(1.24, 2.33)	0.79	0.94	(0.50, 1.78)	1.12	(0.53, 2.39)
Activity restrictions	Never		1.00	1.00		1.00		1.00	1.00		1.00
	Sometimes	1.29	1.34	(1.05, 1.69)	1.39	(1.07, 1.81)	0.87	1.02	(0.53, 1.95)	0.94	(0.47, 1.87)
	Often	0.90	1.07	(0.77, 1.49)	1.19	(0.77, 1.83)	0.48	0.54	(0.19, 1.58)	0.50	(0.16, 1.58)
Back/neck problems	None	1.00	1.00		1.00		1.00	1.00		1.00	
	Disabling	1.99	2.14	(1.62, 2.83)	2.19	(1.62, 2.96)	0.72	0.75	(0.34, 1.69)	1.04	(0.44, 2.43)
Smoking status	Never	1.00	1.00		1.00		1.00	1.00		1.00	
	Past	1.21	1.26	(1.00, 1.60)	1.25	(0.98, 1.60)	0.95	1.10	(0.59, 2.04)	1.32	(0.68, 2.58)
	Current	0.99	0.98	(0.75, 1.28)	0.97	(0.73, 1.30)	0.71	0.64	(0.31, 1.35)	0.68	(0.32, 1.47)
Body weight	Under/normal	1.00	1.00		1.00		1.00	1.00		1.00	
	Overweight	1.07	1.15	(0.91, 1.46)	1.16	(0.91, 1.47)	1.44	1.52	(0.79, 2.94)	1.64	(0.82, 3.24)
	Obese	1.16	1.30	(1.00, 1.69)	1.29	(0.98, 1.69)	0.84	0.85	(0.41, 1.77)	0.78	(0.35, 1.73)
Physical activity (past 3 months)	Active	1.00	1.00		1.00		1.00	1.00		1.00	
	Moderate	0.96	0.97	(0.72, 1.32)	1.03	(0.75, 1.40)	1.05	1.12	(0.48, 2.62)	1.02	(0.42, 2.43)
	Inactive	0.91	0.94	(0.73, 1.21)	0.93	(0.71, 1.21)	1.37	1.49	(0.75, 2.98)	1.21	(0.60, 2.46)
Activity frequency (past 3 months)	Regular	1.00	1.00		1.00		1.00	1.00		1.00	
	Occasional	1.12	1.13	(0.87, 1.48)	1.15	(0.87, 1.52)	0.68	0.67	(0.30, 1.46)	0.60	(0.26, 1.39)
	Infrequent	0.93	1.00	(0.79, 1.27)	0.97	(0.74, 1.26)	1.21	1.27	(0.68, 2.35)	1.11	(0.57, 2.17)
Hospitalization (past year)	None	1.00	1.00		1.00		1.00	1.00		1.00	
	1 or more	0.87	0.86	(0.63, 1.19)	0.85	(0.60, 1.21)	0.38	0.45	(0.16, 1.23)	0.58	(0.19, 1.70)
Prescriptions (past month)	None	1.00	1.00		1.00		1.00	1.00		1.00	
	1 or more	0.97	0.99	(0.79, 1.25)	1.04	(0.82, 1.31)	0.20	0.23	(0.13, 0.42)	0.23	(0.12, 0.44)
Medications (past 2 days)	Per medication	0.94	0.95	(0.89, 1.02)	0.96	(0.89, 1.03)	0.50	0.53	(0.38, 0.72)	0.51	(0.36, 0.70)
Insurance (past year)	Insured	0.81	0.74	(0.51, 1.07)	0.73	(0.49, 1.07)	0.17	0.18	(0.09, 0.38)	0.21	(0.10, 0.44)
Unmet needs (past year)	None	1.00	1.00		1.00		1.00	1.00		1.00	
	Any	1.03	1.04	(0.77, 1.40)	0.99	(0.70, 1.39)	0.85	0.79	(0.36, 1.73)	0.61	(0.21, 1.74)
Regular doctor (past year)	No	1.00	1.00		1.00		1.00	1.00		1.00	
	Yes	0.60	0.61	(0.45, 0.81)	0.59	(0.44, 0.81)	0.09	0.10	(0.05, 0.17)	0.09	(0.05, 0.18)
Health care quality (past year)	Excellent	1.00	1.00		1.00		1.00	1.00		1.00	
	Good	0.94	0.97	(0.79, 1.20)	0.93	(0.74, 1.16)	1.40	1.40	(0.79, 2.46)	1.30	(0.71, 2.38)
	Fair or poor	0.95	1.00	(0.72, 1.39)	0.95	(0.67, 1.35)	1.17	1.11	(0.47, 2.62)	1.09	(0.39, 3.04)
Health care satisfaction (past year)	Very satisfied	1.00	1.00		1.00		1.00	1.00		1.00	
	Somewhat sat.	1.04	1.03	(0.83, 1.29)	0.96	(0.77, 1.21)	1.73	1.57	(0.88, 2.80)	1.31	(0.70, 2.48)
	Not satisfied	1.09	1.12	(0.81, 1.56)	1.03	(0.73, 1.47)	3.35	2.57	(1.26, 5.23)	2.83	(1.26, 6.36)
Quality of MD care (past year)	Excellent	1.00	1.00		1.00		1.00	1.00		1.00	
	Good	0.95	0.97	(0.76, 1.24)	0.94	(0.73, 1.21)	0.46	0.51	(0.20, 1.27)	0.56	(0.20, 1.55)
	Fair or poor	0.69	0.75	(0.47, 1.21)	0.71	(0.43, 1.19)	1.29	1.32	(0.33, 5.27)	1.58	(0.32, 7.97)
Satisfaction of MD care (past year)	Very satisfied	1.00	1.00		1.00		1.00	1.00		1.00	
	Somewhat sat.	0.84	0.83	(0.63, 1.09)	0.77	(0.58, 1.03)	1.89	1.70	(0.68, 4.30)	1.73	(0.64, 4.68)
	Not satisfied	0.83	0.81	(0.48, 1.37)	0.72	(0.41, 1.29)	1.26	1.09	(0.23, 5.22)	0.99	(0.15, 6.27)

* Estimated effects adjusted for age (18–44, 45–64, 65+ years), sex, and race/ethnicity (white only, other/multiple races or ethnicities). ^† ^Estimated effects adjusted for age (18–44, 45–64, 65+ years), sex, race/ethnicity (white only, other/multiple races or ethnicities), education (no high school degree, high school degree or GED, some college, 4-year college degree or certificate), main source of income (employment, other sources), health-related quality of life (Health Utility Index score [continuous]), and health insurance status (insured in past 12 months, not insured in past 12 months).

^‡ ^Odds ratio not shown because of singularities in data for fitting model.

OR = odds ratio; CI = confidence interval; GP = general practitioner or family doctor; DC = Doctor of Chiropractic; HUI = Health Utility Index; CHD = coronary heart disease; MD = medical doctor.

**Table 7:** Crude and adjusted effect estimates of socioeconomic and demographic, quality of life, and health-related factors on seeking in the past 12 months (1) any DC vs. GP care only and (2) DC only vs. both GP and DC care: Results from logistic regression analyses among Canadian respondents from the Joint Canada/US Survey of Health, 2002–03

		Any DC vs. GP Care Only (n = 2882)					DC Only vs. GP+DC Care (n = 448)				
		Adjusted effect estimates					Adjusted effect estimates				
Variable	Category	Crude OR	Model 1* OR	95% CI	Model 2^† ^OR	95% CI	Crude OR	Model 1* OR	95% CI	Model 2^† ^OR	95% CI
Age (Years)	18–44	1.00	1.00		1.00		1.00	1.00		1.00	
	45–64	0.86	0.83	(0.64, 1.07)	0.85	(0.66, 1.11)	0.29	0.29	(0.11, 0.73)	0.34	(0.12, 0.94)
	≥ 65	0.49	0.49	(0.36, 0.67)	0.68	(0.44, 1.04)	0.18	0.17	(0.05, 0.61)	0.18	(0.05, 0.71)
	Per 10 years	0.90	0.90	(0.82, 0.90)	1.00	(0.90, 1.00)	0.60	0.61	(0.45, 0.74)	0.61	(0.45, 0.82)
Sex	Female	1.00	1.00		1.00		1.00	1.00		1.00	
	Male	1.06	1.05	(0.83, 1.31)	1.03	(0.82, 1.30)	4.55	4.65	(2.19, 9.87)	3.63	(1.67, 7.90)
Race/ethnicity	White only	1.00	1.00		1.00		1.00	1.00		1.00	
	Other/multiple	0.65	0.62	(0.44, 0.87)	0.61	(0.42, 0.87)	1.15	1.12	(0.46, 2.76)	1.29	(0.53, 3.16)
Education	No H.S. degree	1.00	1.00		1.00		1.00	1.00		1.00	
	H.S. degree	1.52	1.32	(0.92, 1.90)	1.42	(0.98, 2.06)	0.73	0.48	(0.17, 1.40)	0.61	(0.20, 1.85)
	Some college	1.28	1.09	(0.73, 1.60)	1.19	(0.80, 1.77)	0.66	0.61	(0.22, 1.65)	0.78	(0.26, 2.36)
	4-year degree	1.58	1.40	(0.97, 2.02)	1.52	(1.05, 2.20)	0.29	0.23	(0.08, 0.70)	0.30	(0.09, 1.00)
Marital status	Married	1.00	1.00		1.00		1.00	1.00		1.00	
	Was married	0.77	0.92	(0.68, 1.25)	0.93	(0.67, 1.29)	0.25	0.49	(0.13, 1.86)	0.51	(0.14, 1.84)
	Single	0.87	0.77	(0.56, 1.07)	0.73	(0.52, 1.01)	2.70	1.84	(0.82, 4.10)	1.55	(0.65, 3.70)
Income (adjusted for household size)	Highest quint.	1.00	1.00		1.00		1.00	1.00		1.00	
	Upper middle	0.84	0.81	(0.56, 1.16)	0.80	(0.55, 1.16)	1.43	1.63	(0.51, 5.23)	1.56	(0.50, 4.90)
	Middle	0.71	0.70	(0.48, 1.04)	0.71	(0.47, 1.07)	2.11	2.23	(0.64, 7.79)	1.93	(0.51, 7.25)
	Lower middle	0.79	0.83	(0.57, 1.22)	0.84	(0.56, 1.27)	2.33	2.82	(0.88, 8.97)	2.45	(0.70, 8.59)
	Lowest quint.	0.44	0.49	(0.32, 0.73)	0.49	(0.30, 0.80)	1.76	2.34	(0.64, 8.55)	2.15	(0.46, 9.96)
Main income source	Employment	1.00	1.00		1.00		1.00	1.00		1.00	
	Other	0.62	0.78	(0.55, 1.12)	0.76	(0.53, 1.11)	0.38	0.86	(0.30, 2.45)	0.92	(0.33, 2.52)
HUI	Per 0.1 unit	0.99	0.97	(0.93, 1.02)	0.95	(0.90, 1.00)	1.25	1.12	(0.90, 1.40)	1.12	(0.89, 1.41)
Self-rated health	Excellent	1.00	1.00		1.00		1.00	1.00		1.00	
	Very good	0.93	0.96	(0.71, 1.30)	1.01	(0.74, 1.37)	0.51	0.58	(0.26, 1.29)	0.58	(0.26, 1.30)
	Good	0.76	0.88	(0.63, 1.23)	0.95	(0.67, 1.34)	0.55	0.76	(0.29, 1.98)	0.70	(0.24, 2.03)
	Fair or poor	0.83	1.03	(0.68, 1.56)	1.11	(0.65, 1.87)	0.08	0.11	(0.01, 0.86)	0.09	(0.01, 0.66)
Depression (past year)	1+ episodes	1.06	0.96	(0.64, 1.44)	0.95	(0.61, 1.47)	0.33	0.38	(0.08, 1.73)	0.39	(0.08, 2.04)
Mental health (past year)	1+ visits	1.34	1.25	(0.90, 1.74)	1.23	(0.87, 1.74)	‡	‡		‡	
Emotional problems	Happy in life	1.00	1.00		1.00		1.00	1.00		1.00	
	Not happy	0.75	0.76	(0.56, 1.03)	0.70	(0.50, 0.99)	1.10	1.64	(0.61, 4.40)	2.80	(0.93, 8.44)
Chronic conditions (lifetime history)	Asthma	1.15	1.07	(0.76, 1.52)	1.12	(0.78, 1.61)	0.81	0.67	(0.21, 2.12)	0.81	(0.25, 2.68)
	Arthritis	1.08	1.37	(1.02, 1.83)	1.33	(0.98, 1.82)	‡	‡		‡	
	Hypertension	0.53	0.62	(0.45, 0.86)	0.65	(0.46, 0.91)	0.15	0.26	(0.05, 1.35)	0.30	(0.06, 1.58)
	Emphysema	0.84	0.99	(0.39, 2.52)	1.02	(0.39, 2.72)	‡	‡		‡	
	Diabetes	0.65	0.79	(0.44, 1.42)	0.77	(0.42, 1.42)	0.15	0.25	(0.03, 2.43)	0.25	(0.02, 2.97)
	Heart disease	0.72	0.90	(0.53, 1.50)	0.96	(0.57, 1.61)	0.18	0.33	(0.04, 2.60)	0.46	(0.05, 3.88)
	CHD	0.58	0.72	(0.28, 1.80)	0.74	(0.29, 1.88)	0.66	1.60	(0.25, 10.30)	1.89	(0.18, 20.44)
	Angina	0.42	0.57	(0.28, 1.15)	0.61	(0.29, 1.27)	‡	‡		‡	
	1 or more	0.84	1.03	(0.80, 1.33)	1.05	(0.80, 1.37)	0.14	0.19	(0.07, 0.56)	0.20	(0.06, 0.67)
	Per condition	0.88	0.96	(0.85, 1.08)	0.97	(0.85, 1.10)	0.29	0.34	(0.15, 0.78)	0.36	(0.14, 0.91)
Pain/activity limits	No pain	1.00	1.00		1.00		1.00	1.00		1.00	
	Pain	1.46	1.58	(1.20, 2.07)	1.92	(1.34, 2.76)	0.30	0.38	(0.14, 1.00)	0.30	(0.07, 1.18)
Activity restrictions	Never		1.00	1.00		1.00		1.00	1.00		1.00
	Sometimes	1.33	1.41	(1.09, 1.84)	1.43	(1.08, 1.89)	0.35	0.38	(0.16, 0.92)	0.39	(0.16, 0.96)
	Often	0.96	1.05	(0.73, 1.51)	1.06	(0.66, 1.72)	0.50	0.61	(0.19, 1.95)	0.56	(0.13, 2.54)
Back/neck problems	None	1.00	1.00		1.00		1.00	1.00		1.00	
	Disabling	2.27	2.37	(1.76, 3.19)	2.38	(1.73, 3.29)	0.20	0.18	(0.06, 0.58)	0.19	(0.07, 0.56)
Smoking status	Never	1.00	1.00		1.00		1.00	1.00		1.00	
	Past	1.12	1.15	(0.88, 1.50)	1.20	(0.91, 1.57)	0.23	0.24	(0.08, 0.72)	0.22	(0.07, 0.64)
	Current	1.02	0.94	(0.69, 1.27)	0.96	(0.70, 1.32)	2.61	2.77	(1.29, 5.95)	2.13	(0.97, 4.65)
Body weight	Under/normal	1.00	1.00		1.00		1.00	1.00		1.00	
	Overweight	1.12	1.14	(0.88, 1.48)	1.11	(0.85, 1.44)	0.81	0.60	(0.27, 1.34)	0.55	(0.25, 1.22)
	Obese	0.99	0.97	(0.69, 1.36)	0.97	(0.69, 1.37)	0.46	0.24	(0.07, 0.83)	0.24	(0.07, 0.83)
Physical activity (past 3 months)	Active	1.00	1.00		1.00		1.00	1.00		1.00	
	Moderate	0.91	0.95	(0.70, 1.29)	0.96	(0.70, 1.31)	1.09	1.71	(0.67, 4.38)	1.56	(0.61, 4.02)
	Inactive	0.95	1.02	(0.77, 1.35)	0.99	(0.74, 1.32)	1.60	2.25	(0.93, 5.45)	1.75	(0.71, 4.33)
Activity frequency (past 3 months)	Regular	1.00	1.00		1.00		1.00	1.00		1.00	
	Occasional	1.15	1.16	(0.85, 1.58)	1.17	(0.86, 1.60)	1.09	0.85	(0.28, 2.62)	0.82	(0.27, 2.50)
	Infrequent	0.81	0.91	(0.65, 1.28)	0.81	(0.56, 1.17)	2.29	3.74	(1.62, 8.66)	2.42	(0.89, 6.60)
Hospitalization (past year)	None	1.00	1.00		1.00		1.00	1.00		1.00	
	1 or more	0.70	0.75	(0.51, 1.11)	0.74	(0.49, 1.10)	0.64	0.89	(0.22, 3.53)	1.11	(0.26, 4.70)
Prescriptions (past month)	None	1.00	1.00		1.00		1.00	1.00		1.00	
	1 or more	0.76	0.85	(0.67, 1.09)	0.86	(0.67, 1.10)	0.13	0.19	(0.07, 0.49)	0.21	(0.08, 0.57)
Medications (past 2 days)	Per medication	0.92	0.96	(0.86, 1.07)	0.96	(0.85, 1.08)	0.19	0.25	(0.09, 0.68)	0.26	(0.10, 0.65)
Unmet needs (past year)	None	1.00	1.00		1.00		1.00	1.00		1.00	
	Any	1.06	1.01	(0.72, 1.41)	1.00	(0.70, 1.43)	0.86	0.78	(0.30, 2.05)	1.02	(0.34, 3.08)
Regular doctor (past year)	No	1.00	1.00		1.00		1.00	1.00		1.00	
	Yes	0.79	0.88	(0.60, 1.29)	0.95	(0.65, 1.40)	0.16	0.24	(0.10, 0.56)	0.25	(0.10, 0.63)
Health care quality (past year)	Excellent	1.00	1.00		1.00		1.00	1.00		1.00	
	Good	1.22	1.20	(0.93, 1.55)	1.24	(0.96, 1.60)	2.10	2.21	(0.93, 5.25)	2.08	(0.83, 5.20)
	Fair or poor	1.40	1.39	(0.98, 1.98)	1.36	(0.94, 1.95)	2.49	2.01	(0.70, 5.75)	2.12	(0.68, 6.61)
Health care satisfaction (past year)	Very satisfied	1.00	1.00		1.00		1.00	1.00		1.00	
	Somewhat sat.	1.00	0.97	(0.76, 1.25)	0.99	(0.77, 1.28)	1.69	1.69	(0.73, 3.90)	1.55	(0.65, 3.72)
	Not satisfied	1.38	1.31	(0.93, 1.84)	1.25	(0.88, 1.78)	2.81	2.38	(0.92, 6.16)	1.96	(0.72, 5.32)
Quality of MD care (past year)	Excellent	1.00	1.00		1.00		1.00	1.00		1.00	
	Good	1.24	1.27	(0.96, 1.68)	1.23	(0.92, 1.63)	1.17	1.36	(0.32, 5.82)	1.66	(0.39, 7.06)
	Fair or poor	2.16	2.21	(1.41, 3.47)	2.25	(1.42, 3.58)	1.41	1.36	(0.28, 6.57)	1.47	(0.35, 6.19)
Satisfaction of MD care (past year)	Very satisfied	1.00	1.00		1.00		1.00	1.00		1.00	
	Somewhat sat.	1.17	1.16	(0.87, 1.56)	1.17	(0.87, 1.58)	1.12	1.10	(0.23, 5.33)	1.18	(0.23, 5.98)
	Not satisfied	1.83	1.72	(1.09, 2.71)	1.65	(1.02, 2.66)	1.76	1.56	(0.30, 8.05)	1.51	(0.35, 6.46)

* Estimated effects adjusted for age (18–44, 45–64, 65+ years), sex, and race/ethnicity (white only, other/multiple races or ethnicities).

^†^Estimated effects adjusted for age (18–44, 45–64, 65+ years), sex, race/ethnicity (white only, other/multiple races or ethnicities), education (no high school degree, high school degree or GED, some college, 4-year college degree or certificate), main source of income (employment, other sources), and health-related quality of life (Health Utility Index score [continuous]).

^‡^Odds ratio not shown because of singularities in data for fitting model.

OR = odds ratio; CI = confidence interval; GP = general practitioner or family doctor; DC = Doctor of Chiropractic; HUI = Health Utility Index; CHD = coronary heart disease; MD = medical doctor.
